# Flourishing or Frightening? Feelings about Natural and Built Green Spaces in Singapore

**DOI:** 10.3390/ijerph21030347

**Published:** 2024-03-14

**Authors:** Denise Dillon, Sean T. H. Lee, Eunice W. L. Tai

**Affiliations:** 1 School of Social and Health Sciences, James Cook University, Singapore 387380, Singapore; 2 College of Healthcare Sciences, James Cook University, Douglas 4811, Australia

**Keywords:** experiential feeling states, eudemonia and apprehension, types of green spaces (natural or built), frequency of experience, nature connectedness, trait anxiety

## Abstract

The current study’s aim was to better understand people’s feelings towards different types of natural and built green space environments in the highly urbanized “garden city” of Singapore. We examined which types of green spaces elicited positive (eudemonic) or negative (apprehensive) affective responses. A total of 288 adult residents of Singapore completed a survey that asked them to report their affective states in response to images of 10 locally different environment types and to complete measures of childhood location, frequency of visiting natural/built environments, nature connectedness, and dispositional anxiety, as well as demographic items on age and gender. The 10 green space environment types were mapped onto an experiential state space representing feelings of apprehension and eudemonia in response to specific types of urban green spaces. In terms of a biophilic response, feelings of eudemonia were no different in natural green spaces compared to built green spaces. A higher frequency of experience in specific environments is associated with enhanced feelings of eudemonia in these environments. The findings indicate that people in Singapore can be apprehensive as much in natural green spaces as in built green spaces, and they can also find eudemonic experiences in built green spaces such as roof-top gardens or town parks.

## 1. Introduction

A wealth of research from a variety of disciplines suggests that exposure to natural environments is emotionally beneficial [1,2,3,4,5,6,7,8,9]. These benefits extend to both behavioural and psychophysiological responses relating to less stress and negative feelings, enhanced positive feelings, and improved subjective well-being. Natural environments can also help restore depleted emotional and cognitive resources [10,11,12], such as attentional capacities following directed attention [13]. Green and blue spaces can also reduce psychophysiological stress [14] and boost positive feelings and mood in general populations, e.g., [15], and aging populations [16]. Natural green spaces such as hills and woodlands and natural blue spaces such as lakes and seas have received more positive ratings in experimental studies exploring preferences for and the perceived restorativeness of images of different environment types [9,17,18]. For the purposes of this review, natural green spaces are defined as nature that has developed naturally with minimum human-made interference, while built green spaces refer to urban land covered by vegetation of any kind which has aesthetic and recreational value [19]. 

Two prominent theories of restoration from nature in the literature are attention restoration theory (ART) [20] and psychophysiological stress recovery theory (PSRT) [14]. Clearly, cognition and emotion with respect to reactions to natural environments are interrelated [12], but the present purpose of the review focuses on how people feel during visits to different natural and built green spaces rather than the restorative effects on their cognitive capacities or for stress recovery. The research suggests that positive affective reactions aid in psychological restoration when visiting green spaces; however, responses are not always or not purely positive.

### 1.1. Positive Affective Reactions to Natural Green Spaces

Early research has explored emotional restoration across different environments in both laboratory and field settings [12,15]. In Ulrich’s [14] lab-based research, he presented photographic images of urban scenes (without vegetation or water), nature scenes dominated by vegetation, and nature scenes that included water. The pre–post affective state measures indicated a significantly higher level of sadness in response to viewing urban scenes than that in the other two categories. Similarly, fear arousal was significantly stronger in response to urban environments than to natural scenes dominated by either vegetation or water.

The meta-analytical results from Barton and Pretty [21] showed significant improvements in self-esteem and mood in various habitats and green spaces, including urban green spaces, with the largest difference in green spaces including water. Additionally, Ryan et al. [22] reported significantly higher reports of subjective vitality following exposure to natural (outdoors) rather than urban (or indoor) environments, and this appeared to be associated more with exposure to natural settings rather than being outdoors per se. In their study, subjective vitality was measured using items such as “I feel energized”, reflecting a positive emotion that is associated with a higher level of arousal than other positive states, such as contentment or happiness.

### 1.2. Negative Affective Reactions to Natural Green Spaces

Reactions to the natural environment are not always and not exclusively positive [23,24]. Biophobic responses relate to fear or avoidance of natural stimuli such as snakes and heights, with such responses being evolutionarily advantageous and thus largely innate [25]. Some research has indicated that some natural green spaces, such as wilderness, can evoke negative reactions, e.g., [26,27], due to the notions of mortality associated with subjective assessments of risk [28]. 

### 1.3. Ambivalent Affective Reactions to Natural Green Spaces

Ambivalent responses to natural environments are possible, whereby one can feel or experience both eudemonia and apprehension at one location, and such feelings are often associated with the experience of awe [29]. An example of such an experience could be the mixed feelings of fear and exhilaration in response to climbing a mountain [30], and there are reports of ambivalent responses in different environments as well [31].

### 1.4. The Restorative Potential of Built Green Spaces

A Dutch study [32] showed that residents with more greenery near their homes were less affected by stressful life events than those with less greenery, suggesting that built green space can buffer against stress-induced health impacts. Some research indicates that restorative potential lies in the absence of people in built green spaces, relative to city streetscapes [33,34], while elsewhere it has been demonstrated that the presence of water in built environments enhances their appeal [35,36]. Further, urban green spaces perceived as social and serene were positively associated with perceived restorativeness [37], while self-reported mood and restorative state did not differ for participants who viewed presentations of parklands, tended woodland, or wild woods [38]. However, descriptions of wild woods were found to be more arousing than those of the other two natural depictions. A separate study by White et al. [39] suggests that residents of urban areas with ample green space are relatively happier and are less mentally distressed compared to residents of urban areas with less green space, and Baur [40] suggests that this might be due to an association between nature-based recreation and spirituality. In an Italian study comparing five typologies of urban green space (high to low levels of human-made elements), it was found that the perceived restorativeness was highest in peri-urban green spaces (with lower levels of human-made elements) [41]. Taken as a whole, it appears that there is ample evidence for links between restorative benefits and urban green spaces, but the specific causal factors are yet to be determined [34,42]. 

### 1.5. The Influence of Frequency of Experience

Hinds and Sparks noted that the biophilia hypothesis relates not to learned or conditioned responses but rather to the fact that “specific sensory cues can elicit innate affective or emotional meaning” ([43], p. 71). The same can be said for affective responses to nature and built environments, e.g., [25]. A few studies have suggested a relationship between the frequency of experience in natural environments and its impact on affective reactions [3,26]. Research has also indicated a positive relationship between the frequency of experience in built green spaces and improved subjective psychological well-being [44,45]. In the United Kingdom, Dallimer et al. [44] found that psychological well-being was surveyed to be the lowest for individuals who visited urban green spaces the least often, while individuals who frequently visited urban green spaces reported the highest scores on psychological well-being. Psychological well-being was measured according to feelings of reflection, contemplation, attachment, and continuity with the past. 

Barnes et al. [46] showed that the affective impacts of nature exposure also vary according to the exposure characteristics, such as the duration and frequency of experience in environments and the patterns of human–nature interaction, as well as the unique characteristics of the environment, including biodiversity, landscape type, tree canopy density, and location. These studies thus suggest a possible relationship between the frequency with which individuals experienced natural and built green spaces and their overall psychological well-being.

### 1.6. The Influence of Personality 

The affective impacts of nature exposure vary in terms of their duration and emotional state. They range from short, state-level changes in emotion to longer-lasting shifts in patterns of mood and thoughts [1]. These responses also vary according to individual differences in visitors to these environments [44]. For the current purposes, the influence of personality on affective responses in different types of green spaces has been focused on according to the dimension of trait anxiety rather than state anxiety. This is because the current study accepts the notion that personality has a relatively stable disposition that could reflect an individual’s characteristic pattern of thinking, feeling, and behaving [47].

Neuroticism, a personality dimension, has been found to be highly correlated with trait anxiety [48,49,50]. It is conceivable that certain personality traits such as higher degrees of neuroticism may make an individual more vulnerable to experiencing stress and fatigue [51] and therefore more likely to seek solitude [52]. These individuals are more likely to derive greater benefits in terms of emotional regulation or reduced rumination [2,11]. Stated simply, an individual’s personality may shape their environmental experience and also affect their vulnerability to stress and fatigue. A substantial amount of evidence has indicated that individuals who report lower levels of neuroticism reap noticeably greater levels of positive feelings from natural green space as compared to individuals with higher levels of neuroticism [50]. Conversely, as a counter-hypothesis, Ambrey and Cartlidge [50] suggested that individuals who are more emotionally stable are less likely to feel anxious and worried and hence are more likely to venture out to experience nature. Another study conducted with university undergraduates found that the personality dimension of neuroticism was associated with less time spent in natural environments [53]. Similarly, Sandstrom et al. [54] found that spending more time at home was positively related to neuroticism, suggesting that individuals with higher levels of neuroticism would likely spend less time in outdoor environments (i.e., in both natural and built green spaces).

### 1.7. Background and Context to the Current Study

A comprehensive body of research supports the notion that being close to nature provides people with a sense of calm and overall positive well-being [1,55,56]. While other countries have the luxury of land space and a variety of natural sights and extended vistas, the same cannot be said of Singapore. Most of Singapore’s original natural habitats have been cleared for development, leaving very few natural landscapes, together with a combination of built and manicured green spaces [57,58]. The scarcity of genuinely “wild” environments in high-density cities has led to reports of widespread disconnectedness from nature due to limited access and a subsequent decrease in the quality of nature experiences [59], which makes it important to understand the levels of nature connectedness and their potential influence on perceptions of places. Previous studies in European countries have explored the feelings of eudemonia and apprehension people have towards nature and reported that people generally rated environments such as forests and mountains to be stronger in instilling feelings of eudemonia and apprehension compared to farmlands and parks [26]. In the UK, Hinds and Sparks also found that people who visit nature frequently reported higher eudemonia and lower apprehension than those who rarely did, and those who spent their childhoods in rural locations reported less apprehension compared to those who spent their childhoods in urban locations [26]. The present study aim was to replicate some of Hinds and Sparks’s findings in the relatively less geographically diverse city-state of Singapore.

In Singapore’s highly urbanized environment, exposure to nature is mostly restricted to highly curated green spaces within parks or well-maintained paths through nature reserves. The highest publicly accessible peak in Singapore, Bukit Timah Hill, has an altitude of just 163 metres [60]. It remains to be seen whether the findings reported elsewhere will hold true in cities with fewer opportunities for exposure to natural green spaces and expansive vistas. Multiple studies conducted across various cities, such as Singapore, e.g., [61]; Shanghai, China, e.g., [62]; Sheffield, England, e.g., [44]; and Stockholm, Sweden, e.g., [63], have provided support for the restorative benefits of built green spaces. In contrast, one study conducted in Singapore provided contradicting results [64].

However, there is a need for studies comparing the effects of built and natural areas on health and well-being [65]. Investigations of the restorative benefits to be gained through various attributes of built green spaces [33,66,67] have advanced our understanding in this field of research, but it remains to be determined as to which types of green space provide the best experience for residents or visitors of those spaces [68].

In this study, psychological well-being can be understood from two distinct dimensions: eudemonia and apprehension [69,70,71]. Both concepts are central to the study of well-being. The present study conceptualized eudemonia and apprehension as categories of analysis relating to experience (i.e., subjective experiences and emotions) and functioning (i.e., indices of positive psychological functioning, mental health, and flourishing). First, eudemonia may be described as an experience of positive emotions for a purposeful life [72]. There are some indications that the intrinsic values associated with eudemonia may include feelings of inner peace, empathy, reflection, contemplation, vitality, and a deep appreciation of life [73,74]. For example, the emotion of awe has been said to be associated with feelings of reverence, wonder, and aesthetic pleasure, such as in the experience of oceans, vast trees, and mountains. Apprehension, in the present study, categorizes a general negative emotion, such as feelings of anxiousness, loneliness, and isolation.

As such, this study aims to better understand how Singapore’s residents feel towards various types of natural and built green spaces and to determine the category of green spaces that induces the strongest sense of eudemonia or apprehension, while controlling for trait anxiety. The present study examines the relationship between categories of natural and built green spaces and a set of experiential feeling states representing either eudemonia or apprehension. These fourteen experiential feeling states were derived from the previous exploratory research by Hinds and Sparks [26].

The different types of green spaces featured in the study primarily consist of places often seen and experienced by Singaporeans (e.g., lakes, rooftop gardens, beaches, grassy fields). The present study used a similar method employed by Hinds and Sparks [26], who elicited participants’ feelings about individual landscapes through the use of imagination. To avoid having participants imagining environments not encountered locally, we added photographic accompaniment of environments typically encountered in Singapore. 

### 1.8. Aims and Hypotheses

The current paper aims to complement the existing knowledge to address two key research questions. The first question is, are visits to different types of natural (e.g., woodlands, beaches, and rivers) and built green spaces (e.g., town parks, city streetscapes, rooftop gardens, etc.) in Singapore associated with different affective responses, in particular more positive (indicative of restoration, such as calmness, relaxation, refreshment, and aliveness) than negative feelings (indicative of anxiousness, loneliness, and isolation)? The second question is, are differences in affective responses across different types of natural and built green spaces affected by individual differences in visitors, such as frequency of experience, childhood location, levels of nature connectedness, and trait anxiety? The first aim is to investigate the factorial structure of 14 experiential feeling states. The second aim is to find out whether being in natural green spaces would elicit stronger affective responses (both positive and negative) as compared to being in built green spaces. Building on a partial replication of the methodology employed by Hinds and Sparks [26], this study also aimed to investigate whether the interactive effect of the type of environment (natural green spaces vs. built green spaces) on experiential feeling components (eudemonia vs. apprehension) stands in the highly urbanised but largely green city of Singapore.

Thus, with consideration of the discussed literature and the aims of the current study, the hypotheses were as follows:

**Hypothesis 1** **(H1).** *Based on past research, it is expected that items such as serenity, a sense of awe, contemplation, empathy, aliveness, a sense of freedom, connectedness, and refreshment will load on eudemonia (Component 1) positively. Additionally, items such as isolation, loneliness, and anxiety will load on apprehension (Component 2) positively while items such as a sense of fun, talkativeness, and relaxation will load on apprehension negatively.*

**Hypothesis 2** **(H2).** *When participants view images and imagine being in different types of green spaces (natural or built), it is predicted that the mean scores for the experiential state components of both apprehension (biophobic response) and eudemonia (biophilic response) will be stronger for natural green spaces than for built green spaces.*

**Hypothesis 3** **(H3).** *A significant interaction effect between the types of green space stimuli and frequency of actual experience in natural and built green spaces is hypothesized for both the apprehension and eudemonia levels.*

**Hypothesis 4** **(H4).** *We expected that childhood experience would be significantly predictive of the apprehension and eudemonia levels in response to the natural environment stimuli, such that participants from a rural childhood environment would experience lower levels of apprehension and higher levels of eudemonia when presented with the natural environment stimuli.*

## 2. Methods

We adopted a quasi-experimental approach using a mixed-factorial experiment to analyze the effects of exposure to different types of green spaces (i.e., through viewing photographs) and frequency of experience on positive (eudemonia) and negative (apprehension) affective responses, while controlling for nature connectedness and trait anxiety. The present study is a partial replication and extension of Hinds and Sparks’s study [26], with the addition of the photographic accompaniment and the control variables. The data were collected using an online self-reported survey from May to December 2022. Ethics approval for the study was granted by the James Cook University (JCU) Human Research Ethics Committee (H8502). 

### 2.1. Participants

Self-reported park visitors were recruited through social media and convenience sampling to access both a community sample and students at a private university in Singapore. A total of 288 individuals (181 females, 96 males, 8 non-binary people, 3 null responses) completed the study through an online survey. Descriptive statistics of all the measured variables are summarized in Table 1.

### 2.2. Materials and Measures

The study was conducted in the form of an online survey.

#### 2.2.1. Natural and Built Environments

Despite some debate about whether any environment in the present day can be described as truly natural, e.g., [75], the present study adopted a revised working definition similar to that employed by Abraham et al. [76] in their study of landscape preferences. In the present study, built green spaces refer to “a continuum between nature and designed environments such as parks, gardens, and neighbourhood areas” ([76], p. 59), while the working definition of natural green spaces was retained as any areas or natural settings influenced by minimal or a complete absence of human-made interferences [76]. Such a definition is reasonably consistent with that used by Ulrich as well [12]. 

#### 2.2.2. Photographic Accompaniment of Environments

To prepare the visual stimuli to be used in the survey, a stimulus selection exercise was conducted online via Qualtrics [77]. Student volunteers (*n* = 28) were shown 40 photographs taken and compiled by the first author depicting different environments in Singapore. To ensure that the landscape exposure was not confounded by other factors in the photographs, animals or humans did not feature in the images. The participants categorized each photograph into a list of 10 categories to ensure that there was a common understanding of which types of landscapes were depicted. The 10 categories consisted of beach, forest, grassy field, heritage street, modern city street, rooftop garden, river, town park, wetland, and woodland. Two photographs that were best suited to each category according to the participant responses (i.e., highest frequency of category selection) were used as the stimuli for the study, with a total of 20 photographs selected [Available in the Supplementary Materials].

#### 2.2.3. Experiential Feeling States

The experiential feeling states for each type of environment were measured using the item “Imagine yourself in the environment shown above. To what extent would you feel the following?” The responses were recorded on a 7-point scale ranging from “not at all” (1) to “extremely” (7). The experiential feeling component of eudemonia was computed by taking the mean score of the individual experiential states that loaded heavily on that component for all 10 environments. Then, the mean of the mean scores for each environment for eudemonia categorized under natural environments (viz. beach, forest, river, wetland, woodland) and built environments (viz. grassy field, heritage street, modern city street, rooftop garden, town park) was computed, respectively. The resulting variables were regarded as the mean scores for eudemonia in all the natural and built green spaces, respectively.

Likewise, the experiential feeling component of apprehension was computed in the same way by taking the mean score of three individual experiential states that loaded heavily onto that component for all ten environments. 

#### 2.2.4. Frequency of Experience

The frequency of experience of each type of green space was measured using the item, “On average, how often do you visit or experience the type of environment as the one shown above?” The responses were recorded on a 5-point scale ranging from “never” (1) to “very often” (5). The frequency of experience for each natural and built green space was computed by mean-averaging the frequency of experience for each participant. These variables were regarded as pseudo-independent variables as per Hinds and Sparks [26], whereby the mean scores were converted using a median split procedure (median = 3.8 for natural; median = 3.7 for built). This resulted in a three-level ordinal variable (low, mid, and high) for each.

#### 2.2.5. Childhood Location

The type of location in which participants grew up was measured using one question, “In what sort of location did you spend the majority of your childhood?”, with three possible responses with brief descriptors provided: urban (modernized city, city centre, many buildings with few trees, high traffic), suburban (more greenery than city centre but still developed, outside the main city area, neighbourhood towns, moderate traffic), and rural (mostly greenery, few facilities, low traffic, “kampung” environment). 

#### 2.2.6. Nature Connectedness

To measure nature connectedness, we used the 6-item Nature Connectedness Index (NCI) developed by Richardson et al. [78,79]. The NCI is a reliable and valid scale for populations, whether measured face to face or online. It has demonstrated effectiveness in studies exploring associations with a range of pro-environmental or pro-conservation behaviours and key sustainability issues associated with human disconnection from nature [79]. The six items draw on five pathways to nature connectedness: emotion, beauty, contact, meaning, and compassion. Participants respond using a 7-point scale ranging from “completely agree” (1) to “completely disagree” (7). The raw scores were transformed using a weighted points index ranging from 0 to 100 [79]. Richardson et al. [79] reported a high internal consistency of the measure at an alpha of 0.92, and this proved to be the same for the current sample (α = 0.924). 

#### 2.2.7. Brief State–Trait Anxiety Inventory (STAIT-5)

The short version of the Brief State–Trait Anxiety Inventory, STAI-10 [80], is the revised version of the original inventory developed by Spielberger [81]. The brief STAI consists of 10 items: five assessing state anxiety and five assessing trait anxiety. All the items included in the STAI-10 have been reported to demonstrate good, corrected item-total correlations (0.56–0.73) and excellent reliability (α = 0.86) and internal consistency (α = 0.82) [80]. For the present study, we administered the last five items of the STAI-10 to account for dispositional differences in one’s propensity to experience anxiety (α = 0.90). This allowed for a greater degree of confidence that any potential effects observed were independent of one’s anxious predispositions rather than broadly attributable to differences in the characteristics of the stimuli (i.e., type of green space exposure). Trait items are prefaced by the following guidance: “A number of statements which people have used to describe themselves are given below. Read each statement and then select the number at the end of the statement that indicates how you generally feel.” Responses are recorded on a 4-point scale ranging from “not at all” (1) to “very much so” (4). Trait anxiety was computed by taking the sum of scores on all five items for each participant. Higher scores (minimum = 4; maximum = 20) reflected a higher tendency to experience anxiety and a higher vulnerability to anxiety disorders [82].

### 2.3. Procedure

Participants were provided with a link to the Qualtrics survey [77], which was prefaced by an information page describing the study requirements, duration, and options to leave or consent to continue with the study. Upon agreeing to proceed, the participants were presented with demographic questions regarding age and gender, followed by a series of images depicting 10 types of green spaces in randomized order. Along with each image, participants were asked to imagine themselves in the environment presented. Thereafter, the participants were asked to rate how they would feel in that environment according to 14 experiential feeling states. Next, the participants were asked to rate how frequently they visited the environments presented. Lastly, the NCI and trait anxiety items from the STAI-5 were administered. Participation in the survey took approximately 30 min.

## 3. Results

An a priori power analysis was performed using G*Power 3.1 [83], which indicated that a sample size of 80 participants would be required to achieve an excellent-level criterion of 0.98 for a two-factor principal component analysis model (eudemonia; apprehension) [84]. The a priori sample size calculation indicated that for an F-test, analysis of variance, repeated measures, within-factors analysis, a medium effect size (*f* = 0.25), α = 0.05, power (1 − β) = 0.80, 2 groups, 2 measurements, and a nonsphericity correction of 1, a target sample size of 66 was needed. Lastly, multivariate analysis required a minimum sample size of 158. Principal component factor analysis (varimax rotation) was first conducted to ascertain the factor structure and item loadings of Hinds and Sparks’ [26] 14-item experiential states measure (H1). As summarized in Table 2, our results deviated from the factor structure reported by Hinds and Sparks only in one area: the items “talkative”, “sense of fun”, and “relaxed” loaded positively onto the eudemonia factor as opposed to negatively loading onto the apprehension factor. This occurred despite reverse-coding these positively worded items. Running a confirmatory factor analysis (CFA) specifically for the apprehension factor while including these three afflicted items reiterated a poor model fit: χ^2^ (9) = 514.46, *p* < 0.001, CFI = 0.62, RMSEA = 0.45, 90% C.I. = [0.41, 0.48], SRMR = 0.19. Accordingly, we excluded these items when computing the apprehension scores and included them within our computation of the eudemonia scores instead. The resultant measurement model (as per Table 2) yielded a substantially better model fit: χ^2^ (76) = 647.61, *p* < 0.001, CFI = 0.88, RMSEA = 0.16, 90% C.I. = [0.15, 0.18], SRMR = 0.07.

**Figure 1:**
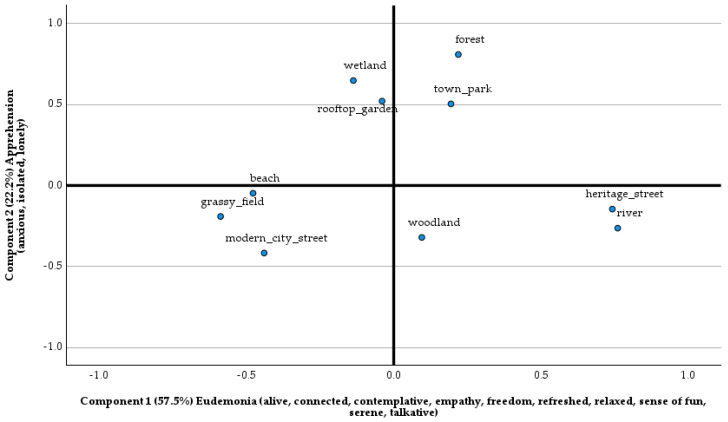
The two-component experiential state space for the 10 environment types as determined using factor scores.

Table 3 summarizes the mean ratings for the experiential states across the 10 types of environments, and Figure 1 represents the two-component space derived from the PCA factor scores. Built environments common to Singapore such as modern city streets and grassy fields were perceived as less experientially apprehensive as well as less eudemonic, whereas heritage streets and river environments were experienced as low in apprehension but high in eudemonia. 

Next, a set of within-samples *t*-tests were conducted to ascertain whether significant mean differences in the apprehension (biophobic response) and eudemonia (biophilic response) scores existed when participants viewed and imagined being in natural versus built green spaces (H2). The results revealed a significant biophobic response, *t* (287) = 12.96, *p* < 0.001, 95% C.I. [0.59, 0.81], such that significantly higher levels of apprehension were observed when participants viewed and imagined being in natural (*M* = 3.68, *SD* = 1.21) as compared to built (*M* = 2.97, *SD* = 1.10) green spaces. However, there were no statistically significant differences in the eudemonia scores when viewing and imagining being in natural (*M* = 3.96, *SD* = 1.09) as compared to built (*M* = 3.93, *SD* = 0.86) green spaces, *t* (287) = 0.52, *p* = 0.61, 95% C.I. [−0.07, 0.12]. These findings held after controlling for the NCI and trait anxiety.

**Figure 2:**
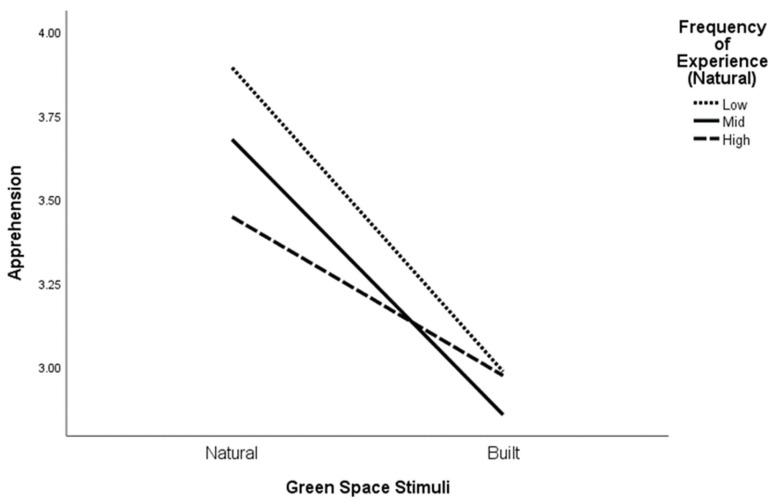
Apprehension levels in response to natural and built green space images according to frequency of past experience in natural environments.

**Figure 3:**
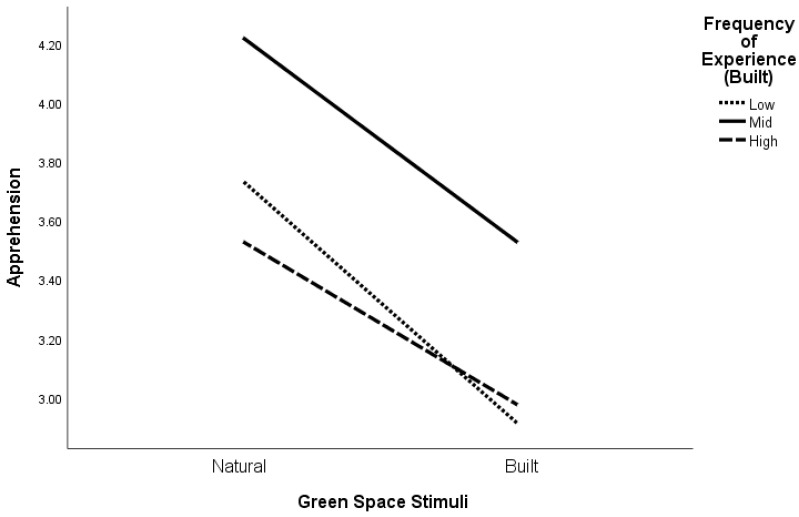
Apprehension levels in response to natural and built green space images according to frequency of past experience in built environments.

To test H3, general linear modeling (repeated measures) was conducted for apprehension and eudemonia separately. For apprehension, the results revealed a significant interaction effect between the type of green space stimuli and the frequency of actual experience in a natural environment (Figure 2 and Table 4), Roy’s Largest Root: *F* (2, 285) = 8.17, *p* < 0.001, as well as between the type of green space stimuli and the frequency of actual experience in built environments (Figure 3 and Table 4), Roy’s Largest Root: *F* (2, 285) = 3.05, *p* = 0.049. These interaction effects held after controlling for the NCI and trait anxiety.

For eudemonia, however, there was no significant interaction observed between the type of green space stimuli and the frequency of actual experience in natural environments, Roy’s Largest Root: *F* (2, 285) = 1.77, *p* = 0.172, as well as for between the type of green space stimuli and the frequency of actual experience in built environments, *F* (2, 285) = 0.79, *p* = 0.455. Controlling for the NCI and trait anxiety did not result in substantive changes to these findings.

To test for H4, general linear modeling (multivariate) was conducted with childhood location specified as the predictor and the apprehension and eudemonia levels in response to the natural environment stimuli specified as the outcome variables. The results revealed a significant main effect of childhood location on both the apprehension, *F* (2, 285) = 3.61, *p* = 0.03, and eudemonia levels, *F* (2, 285) = 3.64, *p* = 0.03. Post hoc multiple comparisons (LSD), however, revealed that the effect on apprehension was not driven by rural versus other childhood locations. Instead, the analyses suggest that it was driven specifically by the urban versus suburban pair (mean difference = 0.35, *p* = 0.03, 95% C.I. [0.04, 0.66]). For eudemonia, the results suggest that the effect was driven specifically by the urban versus rural pair (mean difference = −0.57, *p* = 0.02, 95% C.I. [−1.05, −0.09]). These findings held after controlling for trait anxiety but were all rendered non-significant after controlling for the NCI.

## 4. Discussion

The hypothesis that 14 experiential feeling states would load onto a two-factor model of eudemonia and apprehension was supported (H1). However, not all the individual experiential feeling states loaded onto the components as predicted. Specifically, sense of fun, talkativeness, and relaxation did not load onto apprehension negatively but instead loaded positively onto eudemonia. We postulate that this might have been due to an important difference with respect to how people in Singapore respond to their environments as compared to those in other geographic locations, e.g., [26]. More importantly, the pattern of results described here is consistent with the idea that ambivalent attitudes toward natural and built green spaces are found empirically, e.g., [31]. Nevertheless, the results provide support for the original two factor structure of eudemonia and apprehension proposed by Hinds and Sparks [26]. The feeling state space portrayed in Figure 1 indicates that people in Singapore can be apprehensive as much in natural environments as in built spaces, and they can also find eudemonic experiences in built environments such as rooftop gardens or town parks. 

Our expectation that the feeling states would be stronger in natural green spaces than in built green spaces was supported only in terms of apprehension, indicating a significant biophobic response to natural versus built green spaces amongst the respondents. In terms of a biophilic response, the feelings of eudemonia were no different in the natural green spaces compared to built green spaces. 

With respect to our expectation of an interaction effect between the type of green space and frequency of actual experience in either natural or built green spaces, it appears that feelings of apprehension in natural environments are associated with less direct experiences in such places. Apprehension was overall experienced less in the built environments compared with the natural environments when we consider direct experience in natural environments. This indicates that people feel more anxious, isolated, and lonely in natural environments than in built environments, and this is consistent regardless of how much direct experience they have in either natural or built environments. As for feelings of eudemonia, it appears that experiences were consistent in response to green spaces regardless of whether they were natural or built. 

Predictions of apprehension and eudemonia based on childhood location are more complex, but differences appear to be affected more so by having grown up in urban locations than having grown up in rural or suburban locations. The finding that one’s current level of nature connectedness cancels out this latter effect indicates childhood location is not as strong an influence on a biophilic response as might be supposed. The apprehension levels were highest amongst those who spent their childhood in predominantly urban environments and those who visited green spaces less frequently. However, controlling for nature connectedness and trait anxiety wiped out such effects. 

It seems that we need to be aware that the adage of “feeling at home in nature” might mean different things in various contexts to different people. Fully understanding one’s experience in various environments warrants more than just investigating their environment and affective dimensions—it requires an in-depth look into their aesthetic experience (i.e., what one attends to in their environment), as well as their stimulus-driven and goal-directed attention in an environment [85]. For example, it is usually positively accepted that some form of fear is essential to achieving a “sense of adventure”. A sense of adventure refers to the desire to explore new and unfamiliar places, to want to have new experiences, and to challenge oneself physically and mentally. It is a quality that enables one’s life to be enriched and one’s views to broaden. Without this arguably apprehensive effect, adventurous spirits may never feel fulfilled, leading to a deteriorated sense of wellbeing. Apprehensive states essentially work to help individuals assess the risk and benefits of their actions, heighten their awareness and senses, and even enhance their satisfaction and enjoyment in such a situation. Without this, adventure could lose its meaning and value. 

Singapore has more public green spaces than many other large cities [86]. It might therefore be expected that residents could experience the well-evidenced restorative effects of urban green spaces. However, some speculate an alternative view whereby the persistently green garden city effect might reduce the likelihood of residents being able to gain any further benefits over time [87,88].

### 4.1. Practical and Theoretical Implications

The findings from this study broaden our understanding of people’s psychological responses when occupying natural green spaces. Urban planners can exploit the health-enhancing aspect of specific natural green spaces and improve on built spaces by ensuring they have at least some natural qualities. The findings of this study serve to urge the authorities and urban planners of Singapore to actively prioritize, protect, and strategically plan green spaces in the locations that Singapore citizens frequent. By strategically placing green spaces to allow for or encourage movement rather than placing green spaces in pockets between urban developments, the affective responses to both natural and built green spaces might be maximized.

A major theoretical implication of this study surrounds the interpretation and understanding of the terms eudemonia and apprehension. In this paper, eudemonia is understood as a category of ostensibly positive feelings, while apprehension is regarded as ostensibly negative feelings. However, the conceptual and operational understandings of the two concepts have been debated and challenged [69,71]. Some researchers have described eudemonia as a way of functioning, while apprehension is described as an experience. Others have operationalized the concepts at both the trait and state levels. It is therefore important to consider the scope within which each of the terms is intended, and the breadth of scope should not sacrifice the level of informativeness. For instance, Huta and Waterman (p. 1431) [69] categorize eudemonia and apprehension into four categories: “(a) orientations: values, motives, and goals (the ‘‘why’’ of behaviour), (b) behaviours: behavioural content and activity characteristics (the ‘what’’ of behaviour), (c) experiences: subjective experiences, emotions, and cognitive appraisals, and (d) functioning: indices of positive psychological functioning, mental health, and flourishing” [69,89].

This paper conceptualizes eudemonia and apprehension as part of the latter two categories of experience and functioning. Additionally, the theoretical understanding of what may constitute feelings of eudemonia or apprehension could differ across cultures and contexts [90]. For instance, it appears that people from Eastern cultures experience ambivalent emotions more so than those from Western cultures [91]. Additionally, Schimmack et al. [92] found that the negative correlation between positive affect and negative affect is stronger in individualist as compared to collectivist nations. Therefore, a theoretical implication of the study is the single-faceted operationalization of the concepts of eudemonia and apprehension. Although the present study taps into two categories of analysis, it is worth noting that a thorough understanding of the different categories of analysis can bring about a more comprehensive understanding of eudemonic and apprehensive reactions to various green spaces.

### 4.2. Limitations and Future Research

There are several limitations associated with this study. Firstly, the frequency of experience was measured on an average indicator of how frequently the participants visited an environment. This was rated on a five-point scale ranging from “never” (1) to “very often” (5). However, the duration of each frequency of experience was not measured (e.g., as in, the approximate time spent in a location in minutes or hours). The determination of a time frame is crucial in examining the average frequency of experience in a particular environment. The interpretation of “very often” could be understood by a participant as visiting more than three times a week, while others may understand it as visiting a location daily. Additionally, each count of frequency of experience could have been better explained to participants as having spent more than 30 min in a particular environment. With the improved operationalization of this measure, the study could have been better able to account for a more stringent score being given by participants who may walk past a particular built green space (such as a town park or modern city street) daily.

The next limitation of the study is that it does not touch on the quality of both natural and built environments. Specific environmental nuances, such as differences in air quality and environmental noise between natural and built environments, may provide a noticeable effect on participant’s experience of their environments. These variables undoubtedly provide a more holistic view, yet this is extremely difficult to capture and reproduce experimentally. 

Taking into consideration the limitations and findings derived from this study, future research could still employ the use of the 14 experiential feeling states. However, the addition of a short phrase or a sentence that exemplifies or demonstrates that particular feeling could be added to help standardize participants’ interpretations of the experiential feeling states. For instance, “Being in this environment has caused me to feel rejuvenated/refreshed/anxious etc.”. Further exploration could also ask participants to account for the reasons why they may feel a certain emotion (e.g., I feel empathy or reflective in the mountains because it puts me into a state of introspectiveness, etc.). Given that individuals may express many different reasons for using natural and built green spaces, further qualitative studies could explore some of the differences in motivations for visiting green spaces between high- and low-frequency users. By asking participants to state the motivations for their visits, further studies may be able to establish some reasons for a difference in the effects of affective response based on the motivations behind and purpose of visitations to natural green spaces in Singapore. 

Building on the findings derived from built green spaces, future research could delve into the fine-grained mechanisms that surround the effects of various types of built green spaces in Singapore (e.g., rooftop gardens, town parks, modern city streets, heritage streets, park connectors, vertical greenery on office buildings, and others) on individual experiential feeling states. With scarce land space in Singapore and the rapid development of housing estates across the island, coupled with the need to improve mental health, greater research into these aspects could further build on the suggestion that there could be potential in the restorative benefits of built green spaces to improve the psychological and physical well-being of Singapore’s residents and other city-dwellers.

Achieving a deeper understanding of which types of green space are best for the well-being of urban residents should be extremely beneficial to urban planners. The findings in this field of research will be highly relevant to developing and/or developed cities. Moreover, human–environment interaction has been a topic of increasing research interest and is now recognized as highly relevant to government policy relating to both positive physiological human health and overall psychological well-being. Singapore’s urban planners are tapping into the restorative benefits of nature in their attempts to integrate nature into its urban design [61], such as planting trees along the majority of its roads, building shopping centres that incorporate green spaces and community gardens, and integrating nature directly into housing estates (e.g., rooftop gardens, vertical greenery, etc.).

## 5. Conclusions

As expected, the present findings suggest that there may be a diverse pattern of experiential states associated with different natural and built green spaces. We found that in Singapore, there is a noticeable increase in people’s apprehensive states (i.e., anxious, isolated, lonely) when they are exposed to natural environments in contrast to built green spaces but no noticeable difference in eudemonic states (e.g., connected, refreshed, talkative) in the same environments. Additionally, the frequency of visits to both built and natural environments appears to be influential to the development of both apprehensive and eudemonic feelings in those places, but experiences in childhood locations are not. The current findings indicate that one’s current level of nature connectedness cancels out the effects of childhood location, which suggests that the development of nature connectedness amongst urban dwellers is something to be encouraged further. Since urban dwellers in Singapore experience feelings of apprehension in natural environments, it could be prudent to foster opportunities for them to be outdoors in the types of built green spaces in which they feel less apprehension, such as woodlands and river-side parks. For the current sample, apprehension came in the form of states such as anxiety, isolation, and loneliness rather than fear, so there is cause for further study to test whether specific types of urban green spaces might offer restorative experiences for those experiencing such apprehensive states.

## Figures and Tables

**Table 1:** Descriptive statistics.

	*N*	*M*	*SD*	Range
Age (in Years)	287	28.00	12.54	53
Gender (% Female)	285	62.8%		
Childhood Location (% Urban)	288	62.5%		
Eudemonia (Natural Environment)	288	3.96	1.09	5.35
Eudemonia (Built Environment)	288	3.93	0.86	5.44
Apprehension (Natural Environment)	288	3.68	1.21	5.17
Apprehension (Built Environment)	288	2.97	1.10	4.80
Frequency of Visit to Natural Environments	288	3.68	0.75	3.60
Frequency of Visit to Built Environments	288	3.67	0.47	3.10
Nature Connectedness Index (NCI)	288	50.66	27.26	100
Trait Anxiety	286	2.39	0.86	3.00

**Table 2:** Principal component analysis loadings in our current sample for Hinds and Sparks’ [26] experiential state measure.

	Components	
Experiential States	Eudemonia (57.5%)	Apprehension (22.2%)
Alive	**0.85**	0.04
Connected	**0.90**	0.13
Contemplative	**0.76**	0.35
Empathy	**0.75**	0.38
Freedom	**0.93**	0.08
Refreshed	**0.93**	0.12
Relaxed	**0.92**	0.08
Sense of Awe	**0.85**	0.29
Sense of Fun	**0.90**	0.18
Serene	**0.88**	0.19
Talkative	**0.65**	0.39
Anxious	0.22	**0.88**
Isolated	0.14	**0.93**
Lonely	0.09	**0.92**

Note: higher loadings when comparing between the two factors are boldfaced for each item.

**Table 3:** Mean scores on 14 experiential states in five natural green space environments and five built green space environments.

Natural Green Space Environments	Alive	Anxious	Connected	Contemplative	Empathy	Freedom	Isolated	Lonely	Refreshed	Relaxed	Awe	Fun	Serene	Talkative
beach	3.20	4.86	3.92	3.58	3.52	3.57	3.44	3.86	3.75	3.61	3.75	3.79	3.68	3.52
forest	4.45	4.00	3.90	3.87	3.57	4.13	4.19	3.99	4.15	3.95	4.19	3.79	4.15	3.23
river	3.84	3.09	4.85	4.37	3.39	3.91	4.00	3.70	4.30	3.67	3.52	4.26	4.33	4.06
wetland	5.04	4.16	3.77	3.74	3.72	3.54	4.12	4.05	3.86	4.04	3.99	4.04	3.73	4.06
woodland	3.95	4.27	3.27	5.36	4.83	3.13	4.34	4.37	3.98	4.91	3.57	3.46	4.85	5.03
**Built Green Space Environments**														
grassy field	4.56	4.48	4.77	3.46	5.74	4.74	3.49	4.22	4.23	3.85	4.66	3.82	3.63	4.67
heritage street	3.92	3.70	4.17	4.20	3.94	3.71	4.14	3.25	5.42	4.49	3.40	4.04	4.02	3.70
modern city street	3.29	3.89	4.08	3.55	3.42	3.82	3.22	5.62	4.19	3.57	3.93	3.90	3.57	4.28
rooftop garden	3.94	3.97	3.84	3.56	3.96	3.19	5.20	4.02	3.22	3.79	3.73	3.50	3.97	3.34
town park	4.73	4.60	4.32	4.72	3.33	5.39	4.07	3.53	3.74	3.76	3.53	3.95	3.67	3.49

**Table 4:** Means and standard deviations for childhood location and frequency of experience according to the experiential state components of eudemonia and apprehension in response to natural environment stimuli.

		Experiential State Components	
		Eudemonia *M* (*SD*)	Apprehension *M* (*SD*)
Childhood Location	Urban	3.84 (1.12)	3.82 (1.19)
	Suburban	4.09 (1.03)	3.47 (1.18)
	Rural	4.40 (0.98)	3.31 (1.39)
Frequency of Experience (Natural)	Low	3.84 (1.15)	3.89 (1.20)
	Medium	4.05 (1.31)	3.68 (1.41)
	High	4.07 (1.00)	3.45 (1.16)
Frequency of Experience (Built)	Low	4.02 (1.08)	3.74 (1.13)
	Medium	3.95 (1.14)	4.23 (0.87)
	High	3.90 (1.11)	3.54 (1.30)

## Data Availability

The data presented in this study are openly available on Research Data JCU at https://doi.org/10.25903/jv4a-5261.

## Supplementary Materials

The following supporting information can be downloaded at https://doi.org/10.25903/jv4a-5261. Mean (sd) ratings of apprehension (A, anxious, isolated, lonely) and eudemonia (E, alive, awe, connected, contemplative, empathy, freedom, fun, refreshed, relaxed, serene, talkative) for 10 types of environments.
